# Characterization of the effects of nitrate and tungstate, alone or combined, and *Salmonella* Newport inoculation on rumen microbiota, fermentation, and *Salmonella* survivability *in vitro*

**DOI:** 10.3389/fmicb.2026.1841027

**Published:** 2026-07-23

**Authors:** Robin C. Anderson, Megan R. Shaw, Delila D. Dominguez, Casey N. Johnson, Samat Amat, Jackie M. Kotzur, Patricia J. Baynham, Merritt L. Drewery, Ken J. Genovese, Tawni L. Crippen, Ryan J. Arsenault

**Affiliations:** 1 United States Department of Agriculture/Agricultural Research Service, Southern Plains Agricultural Research Center, Food and Feed Safety Research Unit, College Station, TX, United States; 2 Department of Biological Sciences, St. Edward’s University, Austin, TX, United States; 3 Department of Agricultural Sciences, Texas State University, San Marcos, TX, United States; 4 Department of Animal Sciences, North Dakota State University, Fargo, ND, United States

**Keywords:** *in vitro*, methane, nitrate, rumen microbiota, *Salmonella*

## Abstract

Nitrate consumption by ruminants may enrich nitrate-respiring *Salmonella* in the gut. To test whether tungstate, an inhibitor of nitrate reductase, may prevent nitrate-promoted enrichment of *Salmonella*, ruminal microbes inoculated with or without 10^4^ colony-forming units (CFU)/mL of *Salmonella* Newport were incubated for 26 h under simulated rumen conditions with or without 10 mM nitrate, 100 mM tungstate, or their combination. Results indicated more nitrate was metabolized (*p* < 0.05) by ruminal populations supplemented with nitrate alone than with nitrate and tungstate combined, with means (± standard deviations) being 8.55 ± 0.74 and 3.97 ± 0.67 μmol nitrate/mL, respectively. Nitrite accumulations were affected (*p* < 0.05) by tungstate treatment, achieving 4.96 ± 0.26 and 0.18 ± 0.07 μmol/mL in populations supplemented with nitrate alone or combined with tungstate, respectively, when not inoculated with *S.* Newport and achieving 1.95 ± 0.18 and 0.06 ± 0.01 μmol/mL, respectively, when inoculated with *S.* Newport. *Salmonella* increased (*p* < 0.05) in populations treated with tungstate, alone or combined with nitrate (5.82 ± 0.10 and 5.57 ± 0.15 log_10_ CFU/mL, respectively), compared to controls or nitrate-only supplemented populations (1.63 ± 0.58 and 2.84 ± 0.21 log_10_ CFU/mL, respectively). In populations not inoculated with *S.* Newport, the addition of nitrate, tungstate, or their combination increased (*p* < 0.05) wild-type coliforms by 1.4 to 3.9 log_10_ units compared to untreated controls (2.67 ± 0.18 log_10_ CFU/mL). In *S.* Newport-inoculated populations, tungstate treatment, alone or combined with nitrate, increased (*p* < 0.05) coliforms by 2.8 to 3.2 log_10_ units compared to control and nitrate-only supplemented populations (3.06 ± 0.48 and 3.49 ± 0.06 log_10_ CFU/mL, respectively). Wildtype lactic acid bacteria were enriched by tungstate treatment and nitrate supplementation, alone or combined to 0.2 to 1.1 log_10_ units, compared to untreated controls (6.84 ± 0.03 and 6.68 ± 0.16 log_10_ CFU/mL, respectively). Methane production decreased by 70% (*p* < 0.05) in populations supplemented with nitrate, whether alone or combined with tungstate, compared to untreated or tungstate-only treated populations (29.85 ± 5.05 and 23.08 ± 5.49 μmol methane/mL of incubation fluid, respectively). These results indicate that tungstate treatment marginally decreased nitrate metabolism in the ruminal populations but surprisingly promoted *Salmonella* enrichment.

## 1 Introduction

Foodborne salmonellosis occurs via ingestion of food or water contaminated with one or more of over 2,500 *Salmonella enterica* serovars (Lamichhane et al., 2024). Alternative routes of infection, such as direct person-to-person or animal-to-person contact, may occur (Lamichhane et al., 2024). Additionally, in food-producing animals, vertical transmission or transdermal infection by biting insects has been reported, but these routes are considered minor compared to oral-fecal routes of infection (Edrington et al., 2013; Hanson et al., 2016; Porwollik et al., 2018). Within the animal’s gastrointestinal tract, *Salmonella* may successfully colonize along the gut mucosa, where translocation to the host’s systemic environment can occur (Pullinger et al., 2007). While gut-associated *Salmonella* have traditionally been thought to conserve energy for growth primarily via fermentation, reports indicate that *Salmonella* can conserve energy for growth via anaerobic respiration using oxidized nitrogen compounds as electron acceptors (Lopez et al., 2015; Tiso and Schechter, 2015; Vázquez-Torres and Bäumler, 2016). Host production of reactive nitrogen compounds, orchestrated by inflammation induced by invading pathogens, can promote the accumulation of nitrate in the gut lumen and mucosal microenvironment as well as within systemic tissues such as the liver and spleen (Winter et al., 2013; Worley, 2023). Moreover, dietary nitrate, while generally limiting in the intestinal lumen of ruminants, can serve as an additional source of nitrate for *Salmonella* respiration in the gut, and occurrences of animals consuming heat- or drought-stressed forages enriched in nitrates (Hall, 2018) may be an emerging risk factor for *Salmonella* colonization of ruminants. Similarly, the intentional feeding of methane-reducing nitrate-based feed additives to ruminants may favor the growth and subsequent carriage of *Salmonella* in the bovine rumen (Correa et al., 2017).

As reviewed by Stewart (1988) and Moreno-Vivián et al. (1999), *Salmonella* growth is favored by nitrate respiration in anaerobic environments because energy conserved during proton motive force phosphorylation yields more energy for growth and maintenance than fermentation. Additionally, the consumption of electrons by soluble electron carriers may promote a favorable redox balance within the bacterial cell. Thus, nitrate is a strong inducer of respiratory metabolism, enabling *Salmonella* with respiratory nitrate reductase activity to use nitrate as an effective energy-conserving growth substrate in the animal gut. In many bacteria, including foodborne pathogens such as *Salmonella* and *Escherichia coli*, nitrate respiration is carried out by molybdenum-containing enzymes, including formate dehydrogenase (Fdh) and several nitrate reductase isozymes, such as an inducible membrane-bound nitrate reductase (NarG) and periplasmic nitrate reductase (NapA). These enzymes catalyze the oxidation of formate, or other suitable reducing substrates, with electrons generated being donated to carriers in an electron transport chain that terminates in the reduction of nitrate to nitrite, an intermediate that can be further enzymatically reduced to ammonia by one of several nitrite reductase isozymes (Stewart, 1988; Moreno-Vivián et al., 1999; Zhong et al., 2020). An interesting and potentially practical aspect of the Fdh, NarG, and NapA molybdenum-containing enzymes is that they can be inhibited by tungstate, an analog of molybdate, via incorporation of tungsten into molybdenum-containing enzymes in place of molybdenum, thereby yielding inactive tungsten-containing enzymes (Stewart, 1988; Moreno-Vivián et al., 1999; Zhong et al., 2020). Conversely, an additional nitrate reductase isozyme (NarZ), which primarily contributes to nitrate metabolism during the transition from aerobic to anaerobic conditions or when nitrate levels are low, may also be present in *Salmonella* and *E. coli* and is insensitive to tungstate inactivation (Stewart, 1988; Moreno-Vivián et al., 1999).

Recognizing that respiratory nitrate metabolism is an important virulence attribute for gut pathogens, recent studies have reported the effects of tungstate on nitrate metabolism (Zhu et al., 2018; Yang et al., 2023). For instance, Zhu et al. (2018) hypothesized and subsequently demonstrated that tungstate reduced the competitiveness of select nitrate-respiring Enterobacteriaceae in both *in vitro* culture and experimentally challenged mice. For ruminants, which can be poisoned by consuming high nitrate-containing feedstuffs, tungstate has been studied mainly for its potential application to prevent risks of nitrite poisoning, with few studies examining its effects on the growth of rumen microbes or on *Salmonella* (Korzeniowski et al., 1980, 1981; Prins et al., 1980; Marais et al., 1988; Takahashi et al., 1989). However, a recent report found that despite effectively inhibiting nitrate metabolism, supplementation with 50 or 100 mM tungstate in pure or mixed populations of rumen microbes, respectively, promoted rather than inhibited the growth of experimentally inoculated *Salmonella* during *in vitro* cultivation (Anderson et al., 2026). Growth of indigenous populations of *E. coli* and lactic acid bacteria in ruminal microbiota was also increased, and fermentative activities associated with methane production and nitrite metabolism decreased, suggesting that the enhanced growth associated with tungstate treatment was not limited to, but may have been confounded by, the inoculated *Salmonella*. Accordingly, the objectives of this study were to characterize the effects of nitrate, tungstate, and their combination on experimentally inoculated *Salmonella* and wild-type populations of coliforms and lactic acid bacteria during *in vitro* incubations of rumen microbiota. Additionally, this study examined the effects of nitrate, tungstate, and their combination, as well as the effect of *Salmonella* inoculation on nitrate- and nitrite-metabolizing activity and fermentation during *in vitro* incubations.

## 2 Materials and methods

### 2.1 Bacterial strains

*Salmonella enterica* serovar Newport was used as a challenge strain in this study and was the same strain used in the earlier study of Anderson et al. (2026); this serovar is a bovine isolate acquired from the fecal contents of a cow reared on a contemporarily managed New Mexico dairy (Edrington et al., 2004). The *Salmonella* Newport strain was resuscitated from a -80 °C culture frozen in 20% glycerol. It was routinely maintained by serial transfer at 37 °C over successive 24-h intervals in Tryptic Soy (TS) broth (Becton, Dickinson and Company, Sparks, MD, United States). To facilitate recovery and viable cell count enumeration of the *S.* Newport challenge strain from the mixed population of rumen microbes, the strain, being naturally resistant to novobiocin, was made resistant to 20 μg nalidixic acid/mL via repeated transfer in TS broth supplemented with gradually increasing concentrations of nalidixic acid as reported earlier for *S. typhimurium* (Anderson et al., 2007). The resultant novobiocin- and nalidixic acid-resistant *S.* Newport, used as an inoculum for the *in vitro* incubations with the rumen microbiota, was grown overnight in TS broth supplemented with 20 μg nalidixic acid/mL and 25 μg novobiocin/mL. Recovery and enumeration of viable cells from the challenge *S.* Newport strain was accomplished by plating serial 10-fold dilutions to Brilliant Green (BG) agar (Oxoid Ltd., Basingstoke, Hampshire, England) supplemented with 20 μg nalidixic acid/mL and 25 μg novobiocin/mL. Novobiocin and nalidixic acid were purchased from Sigma-Aldrich (St. Louis, MO, United States).

### 2.2 *In vitro* incubations

Ruminal fluid used in this experiment was collected on the morning of use (0900) from an 11-year-old ruminally-cannulated Jersey steer maintained on pasture consisting predominantly of Bermudagrass. Care and use of the cannulated steer complied with all Animal Care and Use requirements of the Southern Plains Agricultural Research Center (Experimental Animal Protocol #2016017) and the Institutional Animal Care and Use Standard Operating Practices of the Southern Plains Agricultural Research Center (updated April 7, 2023).

Rumen fluid was obtained by immediately straining collected digesta through a nylon paint strainer to remove large particulate material (Leyendecker et al., 2004). The filtrate was collected in a 1,500 mL thermos until it was full and then immediately capped to minimize oxygen exposure. Collected fluid was transported to the lab and prepared for use as inoculum while under a continuous flow of carbon dioxide, made oxygen-free via passage through a heated copper-filled Hungate column (Hungate, 1944). At the lab, the ruminal fluid pH, measured with a pH meter, was 6.18.

Rumen fluid (500 mL) was then mixed with 100 mL of anaerobic dilution solution (Bryant and Burkey, 1953) containing 30 mM sodium formate. The rumen fluid suspension was dispensed (9 mL/tube) under a continuous flow of carbon dioxide to 18 × 150 mm crimp-top tubes preloaded with 0.2 g ground alfalfa and 0.1 mL of water or 0.1 mL of the novobiocin- and nalidixic acid-resistant *S.* Newport culture used as inoculum (grown overnight in TS broth). This inoculum was intended to achieve approximately 10^4^ colony-forming units (CFU) *S.* Newport/mL in the incubation fluid at the start of the experiment. While *Salmonella* numbers are typically much lower (< 10^1^ CFU/mL) in naturally infected animals (Fegan et al., 2005), the higher inoculation amount improves the ability to detect statistically significant effects. The tubes were immediately capped upon the addition of the rumen fluid suspensions. Sodium nitrate (NaNO_3_) and sodium tungstate (dihydrate salt, NaWO_4_^.^2H_2_O) were added by injecting small volumes (≤1.0 mL) of separate 1,000 mM carbon dioxide-flushed stock solutions, or equivalent additions of water to achieve 10 mM nitrate and 100 mM tungstate, as well as their respective combination. From a practical perspective, a daily dose of 8.2 g nitrate ion/kg dry matter intake fed in two equal-sized meals to a 600 kg dairy cow with an estimated 120-liter rumen volume and consuming a daily diet at 3% (18 kg) of its body weight would achieve approximately 10 mM nitrate after each meal. For comparison, similar feeding of a SilvAir-supplemented diet, at 1.6% of the dry matter intake inclusion rate recommended by Cargill (https://www.provimi.eu/uk-silvair; accessed June 22, 2026) and assuming 76% nitrate ion concentration and 84% dry matter content of SilvAir, would yield a ruminal concentration of approximately 12 mM nitrate after consumption of each meal. Conversely, the 100 mM tungstate dose was intentionally administered at a much higher concentration to achieve levels expected to be more than sufficient to interact with the diverse nitrate-metabolizing populations within the rumen. Using the same assumptions as above, the 100 mM tungstate dose would equate to approximately 165 g tungstate ions/kg dietary dry matter per day, which would be impractical in production settings. Injections were administered using 1-mL syringes affixed to 21-gauge needles.

The tungstate dose was selected based on the high dose used by Gates et al. (2003), which resulted in an appreciable inhibitory effect against *E. coli*. The nitrate dose was chosen to effectively induce respiratory nitrate metabolism and serve as an alternative electron acceptor that can, at least partially, accept electrons at the expense of methanogenesis (Anderson and Rasmussen, 1998). Each treatment included six tubes: three tubes receiving no inoculation of *S.* Newport and the remaining three tubes inoculated with *S.* Newport. All tubes were incubated concurrently as batch cultures for 26 h at 39 °C.

### 2.3 Analytical

Gas production was measured after incubation by volume displacement, and gas composition was determined by gas chromatography (Bell et al., 2017). Fluid samples collected at the beginning of incubation (within 30 min of tube closure) and after 6 or 26 h of incubation were frozen until later analysis for accumulations of nitrate, ammonia, and nitrite using colorimetric methods (Chaney and Marbach, 1962; Schneider and Yeary, 1973; Cataldo et al., 1975). Upon thawing, fluid samples (1.5 mL) were centrifuged at 10,000 × *g* for 10 min in a Sorvall Legend Micro17 Centrifuge (Thermo Scientific, Waltham, MA, United States), and the resultant supernatant fluids were analyzed without deproteinization as colored samples achieved intensities that were above the background color of the incubation fluid.

Six volatile fatty acids (VFA), including acetate, propionate, butyrate, isobutyrate, valerate, and isovalerate, were measured from fluid samples collected at the beginning and after the 26-h *in vitro* incubation. Briefly, the samples were mixed using a vortex (VWR^®^ digital vortex mixer, Radnor, PA, United States), centrifuged (clinical 100 laboratory centrifuge, VWR) at 2000 × g for 20 min, and then filtered through a pore size of 0.45 μm to separate the supernatant. The filtered supernatant fluids were analyzed using an Agilent 6,890 N gas chromatograph (Agilent Technologies, Inc., Wilmington, DE, United States) equipped with an FID, a 15 m × 0.53 mm × 0.5 μm fused Supleco^®^, Nukol capillary GC column (Millipore, Burlington, MA, United States) and a 7,683 series auto-injector according to previous methods (Goetsch and Galyean, 1985; Sarker et al., 2018). Fermentation measurements are presented as the difference between initial and 26-h concentrations and thus reflect net accumulations.

Stoichiometric estimates of the amount of hexose fermented and fermentation efficiency during the 26-h incubations, the latter based on the heats of combustion of glucose and the respective VFA (Chalupa, 1977), are presented in Equations 1, 2 below:


$$\begin{array}{l} \text{Hexoses fermented}=½\;\text{acetate}+½\;\text{propionate}+\\ \text{butyrate}+\text{valerate} \end{array}$$ (1)

$$\begin{array}{ccc} \text{Fermentation efficiency} & = & \;(\begin{array}{l} 0.62\text{ acetate}+1.09\text{ propionate}+\\ 0.78\text{ butyrate} \end{array})÷\\ & \; & \left(\begin{array}{c} \text{acetate}+\text{propionate}+\\ \text{butyrate} \end{array}\right)\times 100 \end{array}$$ (2)

Reducing equivalents generated and consumed, expressed as μmol H_2_/mL incubation fluid, were calculated as described by Anderson et al. (2010), except that they were modified to account for electrons generated from the presumed oxidation of 5 mM added formate and to account for electrons consumed during accumulation of measured hydrogen and the catabolism of nitrate and nitrite using the following equations.

1. H_2_ generated via VFA metabolism = 2 acetate + propionate + 4 butyrate + 2 valerate + 2 isovalerate.
2. Total H_2_ generated = H_2_ generated via VFA + H_2_ provided by 5 mM added formate.
3. H_2_ consumed by methane production = 4 methane.
4. H_2_ consumed by VFA production = 2 propionate + 2 butyrate + valerate.
5. H_2_ consumed by nitrate metabolism = 1 nitrate metabolized.
6. H_2_ consumed by nitrite metabolism = 3 nitrite metabolized.
7. Total H_2_ consumption = H_2_ consumed by methane production + H_2_ consumed by VFA produced + H_2_ consumed by nitrate metabolized + H_2_ consumed by nitrite metabolized + hydrogen gas produced.
8. H_2_ recovery = total H_2_ consumed/H_2_ generated × 100%.

Experimentally inoculated *S.* Newport within rumen microbiota populations were enumerated by viable cell count by serially diluting and plating collected fluid on novobiocin- and nalidixic acid-supplemented BG agar. Wild-type coliforms and lactic acid bacteria were enumerated similarly using *E. coli*/coliform or Lactic Acid Bacteria Petrifilm (3M™ Petrifilm™, St. Paul, MN, United States), respectively. Mean ± standard deviation (SD) of populations of *S.* Newport, wild-type coliforms, and wild-type lactic acid bacteria determined via viable cell count of fluid samples collected from three untreated incubation tubes sampled at time 0 were 4.01 ± 0.06, 5.46 ± 0.22, and 7.08 ± 0.011 log_10_ CFU/mL (*n* = 3) and reflect population levels in all tubes before initiation of treatment effects.

### 2.4 Statistical analysis

Treatments were conducted in triplicate, with diverse microbial populations in each incubation tube considered an individual experimental unit that could respond to treatment stimuli independently of the populations in the other tubes. Accumulations of nitrate, nitrite, and rates and amounts of nitrate metabolism were analyzed using a 2 (*S.* Newport and tungstate) × 2 (without or with) factorial design, including only populations that had been treated with nitrate, as concentrations of nitrate and nitrite in non-nitrate-supplemented incubations were at or below our limit of detection (≤ 0.10 μmol/mL). Similarly, log_10_ transformations of *S.* Newport (CFU/mL) enumerated in inoculated populations were analyzed using a 2 (nitrate vs. tungstate) × 2 (with or without) factorial design, including only populations inoculated with this pathogen. Analyses of log_10_ CFU/mL for ammonia, indigenous coliforms, lactic acid bacteria, pH, VFA, and stoichiometric estimates were conducted using a 2 (without or with *S.* Newport) × 2 (without or with nitrate) × 2 (without or with tungstate) factorial design. When significant, means were compared using Tukey’s HSD All-Pairwise Comparisons test at *p* ≤ 0.05. All statistical analyses were conducted using STATISTIX10 Analytical Software (Tallahassee, FL, United States).

## 3 Results

### 3.1 Effects on *Salmonella* Newport, coliforms, and lactic acid bacteria

In rumen microbial populations inoculated with *S.* Newport, a main effect of tungstate (*p* < 0.01) and an interaction between tungstate treatment and nitrate addition (*p* < 0.03) were observed in samples measured after 6 and 26 h of incubation (Table 1). Viable cell counts (log_10_ CFU/mL) of *S.* Newport were more than 0.9 to 3.9 log_10_ units higher in microbial populations treated with tungstate, whether alone or combined with nitrate, than in populations not treated with tungstate (Table 1). Populations supplemented with nitrate alone also harbored more *S.* Newport (log_10_ CFU/mL) than untreated control populations after 26 h of incubation (*p* = 0.02) but not after 6 h of incubation (*p* = 0.62; Table 1).

**Table 1:** Effect of *Salmonella* Newport inoculation during nitrate supplementation (N), tungstate treatment (T), or their combination on select microbial populations during *in vitro* incubation of freshly collected rumen microbiota.

Parameter	Without *S.* Newport inoculation				With *S.* Newport inoculation (S)^1^				SEM	*p*-value						
	None	N^2^	T^3^	Combined	None	N	T	Combined		S	N	T	S*N	S*T	N*T	S*N*T
*Salmonella* Newport (log_10_ CFU/mL)																
After 6 h incubation	–^4^	–	–	–	3.10^a^	3.36^a^	4.21^b^	4.04^b^	0.08	–	0.6161	<0.0002	–	–	0.0264	–
After 26 h incubation	–	–	–	–	1.63^c^	2.84^b^	5.82^a^	5.57^a^	0.18	–	0.0238	<0.0001	–	–	0.0043	–
Total coliforms (log_10_ CFU/mL)																
After 6 h incubation	4.86	4.80	4.94	4.78	4.93	4.93	4.95	4.85	0.06	0.1448	0.0981	0.9567	0.5197	0.4719	0.2964	0.9850
After 26 h incubation	2.67^d^	4.12^b^	6.51^a^	6.57^a^	3.06^cd^	3.49^bc^	6.27^a^	6.31^a^	0.14	0.0824	0.0001	<0.0001	0.0199	0.5467	0.0004	0.0238
Lactic acid bacteria^1^ (log_10_ CFU/mL)																
After 26 h incubation	6.84	7.04	7.91	7.98	6.68	7.04	7.09	7.15	0.05	<0.0001	0.0001	<0.0001	0.2891	<0.0001	0.0046	0.2464

1 S (*Salmonella* Newport) was inoculated to achieve 10^4^ colony-forming units (CFU)/mL in the incubation fluid at the beginning of the incubation period.

2 N (Nitrate) concentrations at the beginning of the incubation period, when administered alone or in combination with tungstate, were 11.36 ± 2.28 μmol/mL (mean ± SD).

3 T (Tungstate) was supplemented as sodium tungstate dihydrate to achieve 100 mM at the beginning of the incubation period.

4 NA, not applicable.

^a,b,c,d^Least squares means within rows with unlike superscripts differ at *p* < 0.05 based on a Tukey HSD All-Pairwise Comparisons Test.

Main effects of *S.* Newport inoculation, nitrate addition, or tungstate treatment, or any of their potential interactions were not observed on wild-type coliforms in populations sampled after 6 h of incubation (*p* ≥ 0.10; Table 1). A main effect of *S.* Newport inoculation was also not observed on wild-type coliform numbers (log_10_ CFU/mL) within the ruminal populations after 26 h of incubation (*p* = 0.08); however, main effects of nitrate addition (*p* < 0.01) and tungstate treatment (*p* < 0.01) were observed on wild-type coliforms after 26 h of incubation (Table 1). In this case, the numbers of wild-type coliforms were 0.5 and 3.1 log units higher in the nitrate-supplemented or tungstate-treated populations than in populations not treated with nitrate or tungstate, with the least squares means for main effects being 4.63 and 3.34 log_10_ CFU/mL, respectively. Interactions between *S.* Newport inoculation and nitrate supplementation, S. Newport inoculation and tungstate treatment, and nitrate supplementation and tungstate treatment were also observed after 26 h of incubation (*p* = 0.02), but not after 6 h (*p* = 0.99; Table 1).

**Figure 1:**
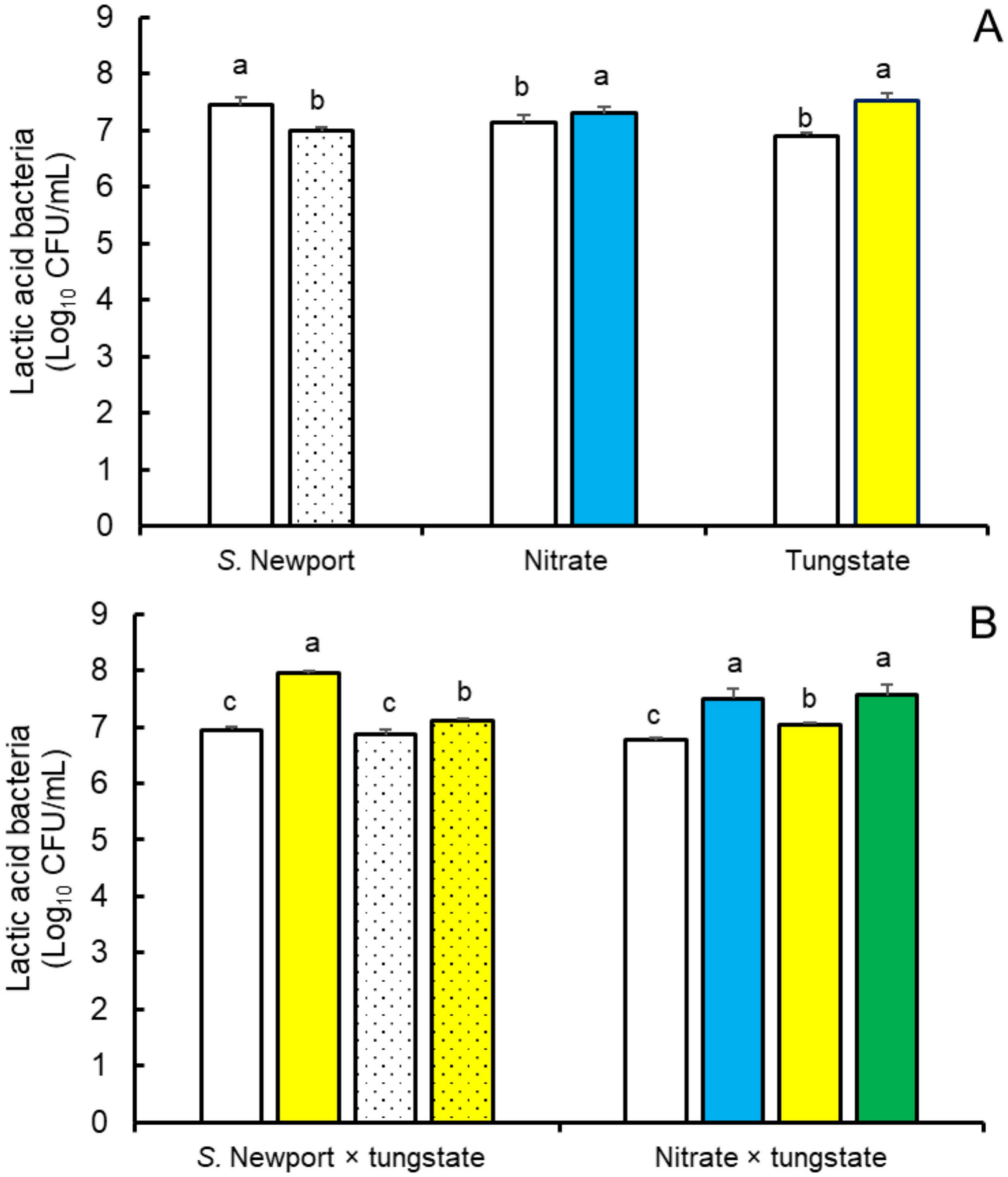
Least squares means ± standard errors for the main effects **(A)** of *S.* Newport inoculation (spot-filled bar), 10 mM nitrate supplementation (blue-filled bar) and 100 mM tungstate treatment (yellow-filled bar) and the interactions **(B)** between *S.* Newport inoculation (spot-filled bars) and 100 mM tungstate supplementation (yellow-filled bars) and the interaction between 10 mM nitrate supplementation (blue-filled bar), 100 mM tungstate treatment and their combination (green-filled bar) on accumulations of lactic acid bacteria during 26-h incubation of mixed populations of ruminal microbes. White-filled bars represent non-treated controls. Least squares means associated with unlike lowercase letters differ at *p* < 0.05 based on a Tukey HSD All-Pairwise Comparisons test.

For wild-type lactic acid bacteria, which were enumerated only in rumen microbiota populations sampled upon initiation and after 26 h of incubation, main effects of *S.* Newport inoculation, nitrate supplementation, and tungstate treatment were observed (*p* < 0.01; Table 1). The numerical differences were modest, with least squares means being 0.46 log_10_ units lower for the *S.* Newport-inoculated populations than for the non-inoculated populations and 0.1 to 0.63 log_10_ units higher for the nitrate-supplemented and tungstate-treated populations than for their respective non-treated populations (Figure 1). Interactions between *S.* Newport inoculation and tungstate (*p* < 0.01), with *S.* Newport inoculation modestly mitigating the effect of tungstate treatment, and between nitrate and tungstate (*p* < 0.01), with tungstate alone being moderately less effective than the incubations containing added nitrate, were also observed (Figure 1). No interaction was observed between *S.* Newport, nitrate addition, and tungstate treatment (*p* = 0.25).

### 3.2 Effects on nitrate, nitrite, and ammonia accumulations

Results from our analysis revealed main effects of *S.* Newport inoculation (*p* < 0.01), tungstate treatment (*p* < 0.01), and their interaction (*p* < 0.01) on rates of nitrate metabolism by the nitrate-containing microbiota, with rates increased in populations inoculated with *S.* Newport and decreased in populations supplemented with tungstate (Table 2). Main effects of *S.* Newport inoculation (*p* ≥ 0.36) or interactions between *S.* Newport inoculation and tungstate treatment (*p* ≥ 0.10) were not observed in the amounts of nitrate catabolized after 6 or 26 h incubation (Table 2). A main effect of tungstate treatment was not observed on amounts of nitrate consumed after 6 h of incubation (*p* = 0.55). However, it was after 26 h of incubation (*p* < 0.01), with 47% less nitrate being metabolized by the nitrate-containing populations treated with tungstate than the non-tungstate-treated nitrate-containing populations (3.97 vs. 8.55 μmol/mL, respectively). A main effect of tungstate (*p* < 0.01) but not of *S.* Newport inoculation (*p* = 0.26) or an interaction between *S.* Newport inoculation and tungstate treatment (*p* = 0.26) was observed on nitrite accumulations after 6 h of incubation, with accumulations being higher for populations incubated without added tungstate (0.97 vs. 0.03 μmol/mL, respectively). Main effects of *S.* Newport inoculation (*p* < 0.01), tungstate treatment (*p* < 0.01), and their interaction (*p* < 0.01) were observed on residual accumulations of nitrite measured at the end of the 26-h incubation (Table 2). In this case, nitrite accumulation in the nitrate-containing populations was highest in those not inoculated with *S.* Newport, intermediate in those inoculated with *S.* Newport but not treated with tungstate, and lowest in nitrate-containing populations treated with tungstate, regardless of whether inoculated or not with *S.* Newport (Table 2).

**Table 2:** Effect of *Salmonella* Newport inoculation during nitrate supplementation (N), tungstate treatment (T), or their combination on accumulations of select nitrogen compounds during *in vitro* incubation of freshly collected rumen microbiota.

Parameter	Without *S.* Newport inoculation				With *S.* Newport inoculation (S)^1^				SEM	*p*-value						
	None	N^2^	T^3^	Combined	None	N	T	Combined		S	N	T	S*N	S*T	N*T	S*N*T
Rate of nitrate metabolism ( μ mol/mL per h)	–^4^	0.33^a^	–	0.11^c^	–	0.32^a^	–	0.17^b^	0.007	0.0070	–	<0.0001	–	0.0009	–	–
Nitrate catabolized after 6 h (μmol/mL)^5^	–	2.70	–	2.61	–	2.28	–	1.12	0.980	0.3589	–	0.5409	–	0.5993	–	–
Nitrate catabolized after 26 h (μmol/mL)	–	8.75	–	3.47	–	8.36	–	4.45	0.370	0.4431	–	<0.0001	–	0.1014	–	–
Nitrite accumulation after 6 h (μmol/mL)	–	0.89	–	0.02	–	1.05	–	0.02	0.068	0.2580	–	<0.0001	–	0.2580	–	–
Nitrite accumulation of after 26 h (μmol/mL)	–	4.96^a^	–	0.18^c^	–	1.95^b^	–	0.63^c^	0.026	<0.0001	–	<0.0001	–	<0.0001	–	–
Nitrite consumed after 6 h (μmol/mL)^6^	–	1.86	–	2.59	–	1.45	–	1.12	0.957	0.3531	–	0.8404	–	0.5966	–	–
Nitrite consumed after 26 h (μmol/mL)	–	3.79	–	3.29	–	6.41	–	4.41	0.382	0.0012	–	0.0113	–	0.0838	–	–
Nitrite consumed as a % of nitrate catabolized after 6 h	–	52	–	99	–	31	–	67	24.7	0.3054	–	0.1316	–	0.8217	–	–
Nitrite consumed as a % of nitrate catabolized after 26 h	–	43^c^	–	95^a^	–	77^b^	–	99^a^	1.6	<0.0001	–	<0.0001	–	0.0002	–	–
Ammonia accumulation after 26 h (μmol/mL)	4.54	5.34	3.66	4.43	5.69	6.15	5.18	6.74	0.260	<0.0001	0.0002	0.0322	0.5463	0.0221	0.1646	0.1442

1 S (*Salmonella* Newport) was inoculated to achieve 10^4^ colony-forming units (CFU)/mL in the incubation fluid at the beginning of the incubation period.

2 N (Nitrate) concentrations at the beginning of the incubation period, when administered alone or in combination with tungstate, were 11.36 ± 2.28 μmol/mL (mean ± SD).

3 T (Tungstate) was supplemented as sodium tungstate dihydrate to achieve 100 mM at the beginning of the incubation period.

4 NA, Not applicable.

5 Least squares means for amounts of nitrate catabolized were calculated as the difference between amounts of nitrate measured at time 0 and after 26 h incubation.

6 Least squares means for amounts of nitrite produced were calculated as the difference between amounts of nitrate catabolized and residual nitrite measured at the end of the 26-h incubation.

^a,b,c^Least squares means within rows with unlike superscripts differ at *p* < 0.05 based on a Tukey HSD All-Pairwise Comparisons Test.

Because the amounts of nitrate catabolized are stoichiometrically equivalent to the amounts of nitrite produced, the difference between amounts of nitrate produced and residual accumulation of nitrite yields an estimate of the amounts of nitrite consumed during further metabolism. Analysis of nitrite consumption after 6 h of incubation revealed no main effects of *S.* Newport inoculation (*p* = 0.35), tungstate treatment (*p* = 0.84), or their interaction (*p* = 0.60). However, after 26 h of incubation, main effects of *S.* Newport inoculation (*p* < 0.01) and tungstate treatment (*p* = 0.01), but not their interaction (*p* = 0.08), were observed on amounts of nitrite consumed (Table 2). Regarding the main effect of *S.* Newport inoculation, 24% more nitrite was consumed in populations inoculated with vs. those without *S.* Newport (5.41 vs. 3.54 μmoL/mL, respectively). Conversely, the main effect means for the effect of tungstate revealed populations treated with tungstate consumed 34% less nitrite than those not treated (3.85 vs. 5.10 μmol/mL, respectively).

For ammonia accumulations during the 26-h incubation, all populations, including populations not treated with nitrate, were analyzed. Results revealed main effects of *S.* Newport inoculation (*p* < 0.01), nitrate addition (*p* < 0.01), or tungstate treatment (*p* = 0.03) as well as an interaction between *S.* Newport inoculation and tungstate treatment (*p* = 0.02), but no interaction between *S.* Newport and nitrate (*p* = 0.55) or a three-way interaction (*p* = 0.14; Table 2). Least squares means for ammonia accumulation from the *S.* Newport inoculation by tungstate treatment interaction were highest for the *S.* Newport-inoculated populations treated with tungstate (5.96 μmol/mL) and without tungstate (5.92 μmol/mL). The means were intermediate for the non-tungstate-treated population not inoculated with *S.* Newport and lowest in the *S.* Newport-inoculated population treated with tungstate (4.94 and 4.05 μmol/mL, respectively).

### 3.3 Effects on fermentation

**Figure 2:**
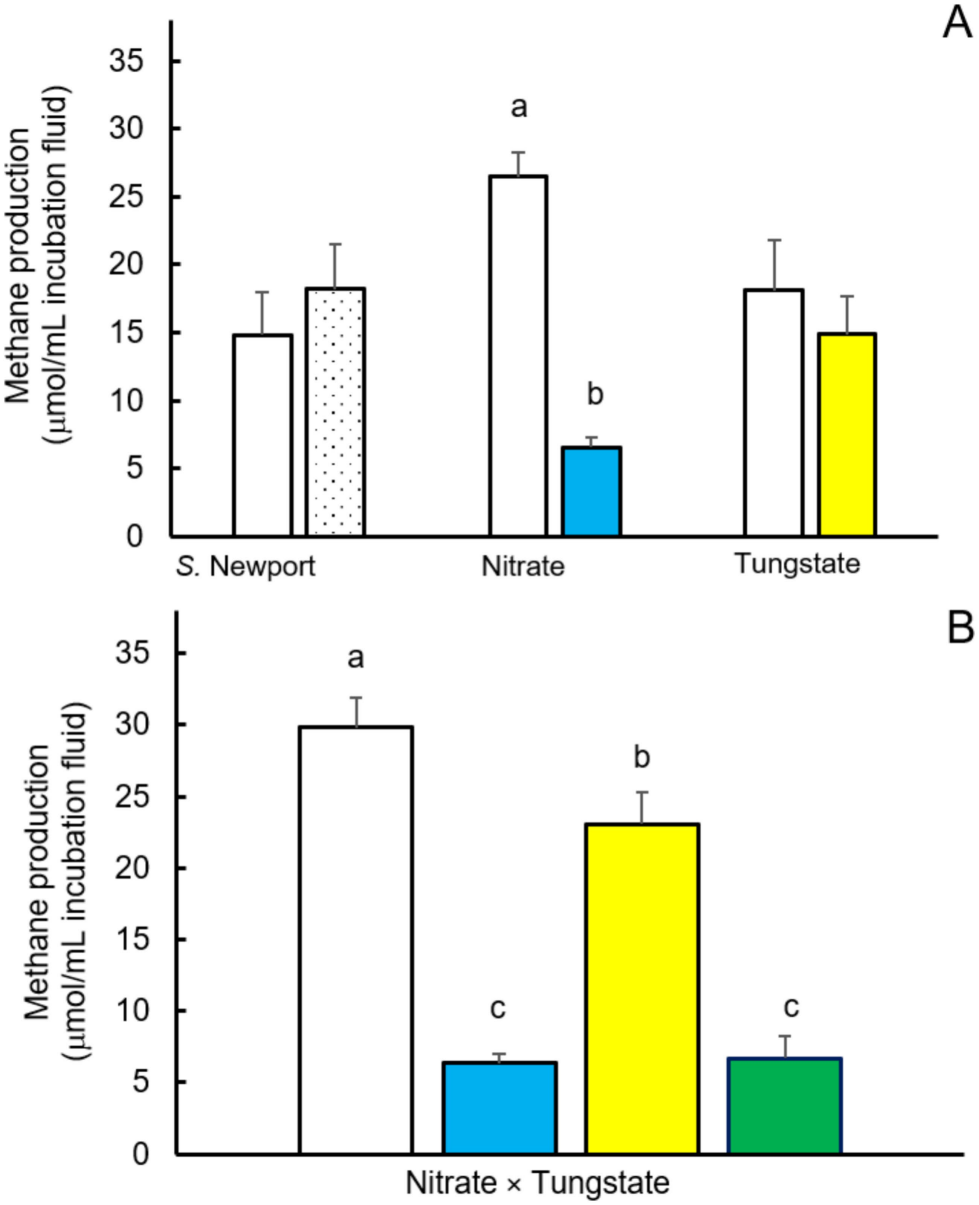
Least squares means ± standard errors for the main effects **(A)** of *S.* Newport inoculation (spot-filled bar), 10 mM nitrate supplementation (blue-filled bar) and 100 mM tungstate treatment (yellow filled bar) and the interaction **(B)** between 10 mM nitrate supplementation (blue filled bars) and 100 mM tungstate treatment (yellow-filled bars for tungstate alone or green-filled bar when combined with nitrate on the production during 26-h incubation of mixed populations of ruminal microbes). White-filled bars represent non-treated controls. Least squares means associated with unlike lowercase letters differ at *p* < 0.05 based on a Tukey HSD All-Pairwise Comparisons test.

Results from analysis of headspace and fluids sampled during incubation of rumen microbiota populations are presented in Table 3. A main effect of nitrate supplementation was observed on total gas produced (*p* < 0.01), with means for the nitrate-supplemented populations being 34% lower than those for the populations not supplemented with nitrate (14.8 vs. 9.8 mL of gas volume fluid, respectively). A main effect of nitrate supplementation (*p* < 0.01) but not of *S.* Newport inoculation (*p* = 0.06) or tungstate treatment (*p* = 0.07) was observed on methane production (Table 3). An interaction between nitrate and tungstate treatments was observed (*p* = 0.05; Table 3 and Figure 2). In the case of the nitrate supplementation by tungstate treatment interaction, least squares means were dramatically lower in populations treated with tungstate alone than in populations supplemented with nitrate, whether alone or combined with nitrate (Figure 2). Other effects or interactions were not observed on total volume of gas produced or on methane production (*p* ≥ 0.14).

**Table 3:** Effect of *Salmonella* Newport inoculation during nitrate supplementation (N), tungstate treatment (T), or their combination on selected fermentation characteristics during *in vitro* incubation of freshly collected rumen microbiota.

Parameter	Without *S.* Newport inoculation				With *S.* Newport inoculation (S)^1^				SEM	*p*-value						
	None	N^2^	T^3^	Combined	None	N	T	Combined		S	N	T	S*N	S*T	N*T	S*N*T
Fermentation characteristics^4^																
Total gas (mL)	16.00	10.33	13.33	8.33	15.33	8.00	14.33	12.33	1.860	0.7088	0.0016	0.8031	0.8031	0.1478	0.2708	0.3881
CH_4_ (μmol/mL)	27.50	5.41	22.61	3.75	32.21	7.35	23.56	9.66	2.370	0.0606	<0.0001	0.0732	0.7443	0.9793	0.0499	0.2668
H_2_ (μmol/mL)	0.05	0.06	0.01	0.07	0.14	0.12	0.11	0.10	0.026	0.0015	0.5407	0.3462	0.2411	0.8634	0.4679	0.6606
C2 (μmol/mL)	43.52	37.50	33.06	35.04	33.02	25.08	25.22	32.96	4.320	0.0161	0.7332	0.3087	0.7575	0.3034	0.0704	0.5382
C3 (μmol/mL)	14.07	9.97	10.68	10.99	7.88	9.74	7.36	8.64	0.972	0.0005	0.8185	0.1653	0.0227	0.7908	0.1817	0.0891
C4 (μmol/mL)	5.87^ab^	1.49^d^	4.69^abc^	2.87^cd^	5.40^ab^	4.32^bc^	6.83^a^	5.10^abc^	0.467	0.0001	<0.0001	0.0871	0.0205	0.1448	0.1665	0.0278
iC4 (μmol/mL)	0.94	0.66	0.84	0.63	0.72	0.62	0.87	0.81	0.036	0.6502	<0.0001	0.0406	0.0085	0.0002	0.4212	0.6689
C5 (μmol/mL)	1.64^abc^	1.59^bc^	1.58^bc^	1.83^ab^	1.52^c^	1.88^a^	1.65^abc^	1.82^ab^	0.055	0.1350	0.0003	0.1426	0.0510	0.4445	0.4847	0.0049
iC5 (μmol/mL)	1.15^de^	0.66^f^	1.14^de^	0.97^e^	1.40^bc^	1.24^cd^	1.92^a^	1.62^b^	0.050	<0.0001	<0.0001	<0.0001	0.1606	0.0007	0.2341	0.0062
Sum (μmol/mL)	67.20	51.88	52.00	52.33	49.95	42.88	43.86	50.96	5.659	0.0401	0.3641	0.4371	0.3622	0.3114	0.0810	0.9772
pH	5.69^b^	5.92^ab^	5.77^ab^	6.33^ab^	5.70^b^	6.35^a^	5.92^ab^	6.01^ab^	0.132	0.4843	0.0008	0.3416	0.9018	0.1267	0.5398	0.0300

1 S (*Salmonella* Newport) was inoculated to achieve 10^4^ colony-forming units (CFU)/mL in the incubation fluid at the beginning of the incubation period.

2 N (Nitrate) concentrations at the beginning of the incubation period, when administered alone or in combination with tungstate, were 11.36 ± 2.28 μmol/mL (mean ± SD).

3 T (tungstate) was supplemented as sodium tungstate dihydrate to achieve 100 mM at the beginning of the incubation period.

4 C2, Acetate; C3, propionate; C4, butyrate; iC4, isobutyrate; C5, valerate; iC5, isovalerate; Sum, sum total of all acids.

^a,b,c,d.e,f^Least squares means within rows with unlike superscripts differ at *p* < 0.05 based on a Tukey HSD All-Pairwise Comparisons Test.

A main effect of *S.* Newport inoculation was observed on accumulations of hydrogen within the microbial populations (*p* ≤ 0.01) such that accumulations were higher for populations inoculated with *S.* Newport (average 0.12 μmol hydrogen/mL incubation fluid) than those not inoculated with *S.* Newport (average 0.05 μmol hydrogen/mL incubation fluid) but main effects of nitrate supplementation, tungstate treatment or any possible interactions were not observed (*p* ≥ 0.24; Table 3).

**Figure 3:**
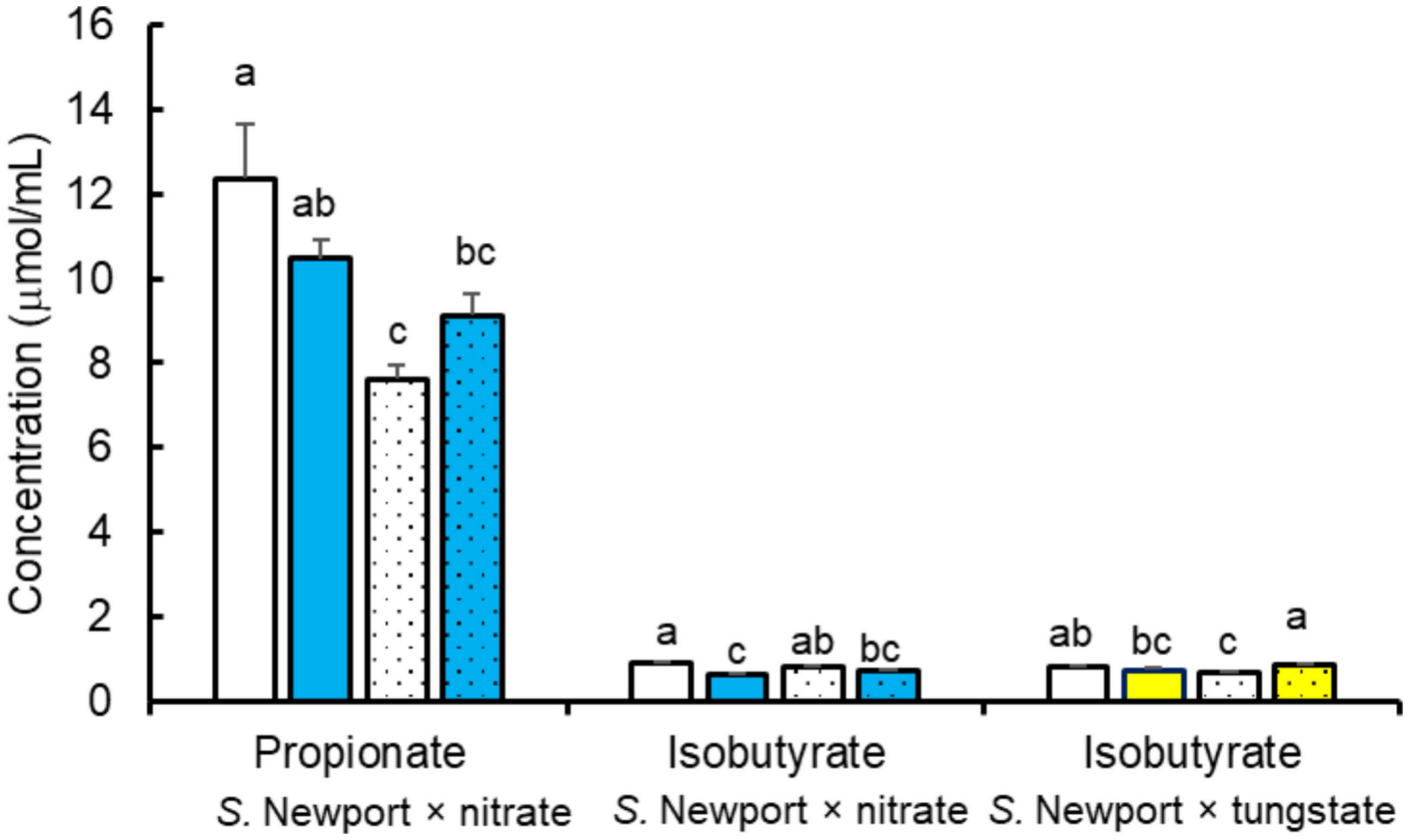
Least squares means ± standard errors for the interactions between *S.* Newport inoculation (spot-filled bars) and 10 mM nitrate supplementation (blue-filled bars) for net accumulations of propionate and isobutyrate and between *S.* Newport inoculation and 100 mM tungstate treatment (yellow-filled bars) for net accumulations of isobutyrate during 26-h incubation of mixed populations of ruminal microbes. White-filled bars represent non-treated controls. Least squares means associated with unlike lowercase letters differ at *p* < 0.05 based on a Tukey HSD All-Pairwise Comparisons test.

With respect to fermentation acid production, a main effect of *S.* Newport inoculation was observed on net production of acetate (C2; *p* = 0.02), with *S.* Newport-inoculated populations producing 22% less acetate than populations that were not inoculated with *S.* Newport (29.07 vs. 37.28 μmol/mL, respectively). Main effects of nitrate supplementation, tungstate treatment, or any possible interactions were not observed for acetate production (*p* ≥ 0.30). A main effect of *S.* Newport inoculation (*p* ≤ 0.01) as well as an effect of *S.* Newport inoculation by nitrate addition interaction (*p* = 0.02), but not of nitrate by itself (*p* = 0.82), was observed on propionate production (C3; Table 3), with least squares means for populations supplemented with nitrate and inoculated with *S.* Newport being lower acetate than populations not supplemented with nitrate or inoculated with *S.* Newport (Figure 3). Main effects of *S.* Newport inoculation (*p* ≤ 0.01) and of nitrate supplementation (*p* ≤ 0.01) were observed on butyrate production (C4; Table 3), with main effect means being higher for *S.* Newport-inoculated populations than those not inoculated (5.41 vs. 3.73 μmol/mL, respectively) and lower for nitrate-supplemented populations than populations not supplemented with nitrate (3.73 vs. 5.41 μmol/mL, respectively). An interaction between *S.* Newport inoculation and nitrate addition (*p* = 0.02), as well as a three-way interaction between *S.* Newport inoculation, nitrate addition, and tungstate treatment (*p* = 0.03), was also observed on butyrate production (Table 3). In the latter case, butyrate production was appreciably lower in nitrate-supplemented populations not inoculated with *S.* Newport than in the populations inoculated with *S.* Newport.

For less abundant fermentation acids, a main effect of *S.* Newport inoculation was observed on isovalerate production (iC5; *p* ≤ 0.01; Table 3), with main effect means higher for *S.* Newport-inoculated populations than those not inoculated with *S.* Newport (1.55 vs. 0.98 μmol/mL, respectively). Main effects of *S.* Newport inoculation were not observed on isobutyrate (iC4) or valerate (C5) production (*p* ≥ 0.14; Table 3). A main effect of nitrate supplementation was observed on the production of isobutyrate, valerate, and isovalerate (*p* ≤ 0.01; Table 3) with respective main effect means being 0.85, 1.60, and 1.14 μmol/mL for populations not supplemented with nitrate and 0.68, 1.78, and 1.12 μmol/mL for the nitrate-supplemented populations. Main effects of tungstate treatment were observed in isobutyrate and isovalerate (*p* ≤ 0.04) production, with main effect means of 0.73 and 1.11 μmol/mL for populations not treated with tungstate and 0.79 and 1.14 μmol/mL for the tungstate-treated populations, respectively. Interactions between *S.* Newport inoculation and nitrate addition (*p* ≤ 0.01) as well as between *S.* Newport inoculation and tungstate treatment (*p* ≤ 0.01) were observed on isobutyrate production (Figure 3), as well as on the production of valerate and isovalerate (Table 3), although differences between the least squares means were small and likely inconsequential. For valerate and isovalerate production, a three-way interaction between *S.* Newport inoculation, nitrate supplementation, and tungstate treatment was observed (*p* ≤ 0.01; Table 3).

A main effect of nitrate supplementation was observed on the pH measured in the populations at the end of the 26-h incubation (*p* ≤ 0.01), but main effects of *S.* Newport inoculation or tungstate treatment were not observed (*p* ≥ 0.34; Table 3). A three-way interaction between *S.* Newport inoculation, nitrate supplementation, and tungstate treatment was observed (*p* = 0.03), but the mean differences did not exceed 0.7 pH units.

Main effects of *S.* Newport inoculation, nitrate supplementation, or tungstate treatment were not observed, nor were any interactions observed on stoichiometric estimates of hexoses fermented (C6; *p* ≥ 0.09; Table 4). Similarly, main effects of *S.* Newport inoculation, nitrate supplementation, or tungstate treatment were not observed on fermentation efficiency (*p* ≥ 0.83). There was an interaction between *S.* Newport inoculation and nitrate supplementation, as well as a three-way interaction between S. Newport inoculation, nitrate supplementation, and tungstate treatment (*p* = 0.04). However, the means differed by no more than 4% and likely reflect little, if any, physiological relevance (Table 4). Estimates of the amounts of reducing equivalents generated during the 26-h incubation, expressed as μmol H_2_ equivalents/mL of incubation fluid, ranged from 88.42 to 135.20 but were not affected by *S.* Newport inoculation, by nitrate supplementation, tungstate treatment or their possible interactions (*p* ≥ 0.08; Table 4).

**Table 4:** Effect of *Salmonella* Newport inoculation during nitrate supplementation (N), tungstate treatment (T), or their combination on stoichiometric estimates of hexose fermentation, fermentation efficiency, and hydrogen balance during *in vitro* incubation of freshly collected rumen microbiota.

Parameter	Without *S.* Newport inoculation				With *S.* Newport inoculation (S)^1^				SEM	*p*-value						
	None	N^2^	T^3^	Combined	None	N	T	Combined		S	N	T	S*N	S*T	N*T	S*N*T
*Stoichiometric estimates*																
C6 fermented (μmol/mL)	36.31	26.82	28.14	27.71	27.38	23.61	24.77	27.72	3.040	0.0903	0.2297	0.5115	0.3052	0.3229	0.0850	0.7885
Fermentation efficiency %	74.15^ab^	72.04^b^	74.01^ab^	73.52^ab^	71.92^b^	75.90^a^	73.59^ab^	72.44^ab^	0.724	0.9509	0.9107	0.8292	0.0176	0.1448	0.1058	0.0446
*Hydrogen balance (μmol H_2_/mL)*																
H_2_ generated																
Via formate	5.00	5.00	5.00	5.00	5.00	5.00	5.00	5.00	–^4^	–	–	–	–	–	–	–
Via VFA	130.20	95.45	101.02	198.15	101.38	83.42	92.25	101.86	11.215	0.1672	0.1666	0.5960	0.3699	0.2760	0.0793	0.8939
Total H_2_ generated	135.20	100.45	106.02	103.15	106.38	88.42	97.25	106.86	11.215	0.1672	0.1666	0.5960	0.3699	0.2760	0.0793	0.8939
H_2_ consumed																
In methane	109.98	21.65	90.45	15.01	128.83	29.40	94.22	38.65	9.479	0.0611	<0.0001	0.0726	0.7478	0.9759	0.0503	0.2649
In hydrogen	0.05	0.06	0.01	0.07	0.14	0.12	0.11	0.10	0.026	0.0015	0.5407	0.3462	0.2411	0.8634	0.4679	0.6606
In VFA	41.53	24.51	32.22	29.54	28.10	30.00	30.02	29.31	2.898	0.2197	0.0374	0.7249	0.0210	0.5185	0.1755	0.0566
In nitrate metabolized	–	8.75	–	3.47	–	8.36	–	4.46	0.370	0.4431	–	<0.0001	–	0.1014	–	–
In nitrite metabolized	–	11.38	–	9.87	–	19.24	–	13.23	1.145	0.0012	–	0.0113	–	0.0838	–	–
Total H_2_ consumed	151.56	66.35	122.68	57.96	157.07	87.12	124.35	85.75	10.916	0.0903	<0.0001	0.0348	0.1977	0.9220	0.1132	0.7277
H_2_ recovery (%)	112.1	66.1	115.7	56.2	147.6	98.5	127.9	80.2	13.12	0.0141	<0.0001	0.2194	0.6387	0.3887	0.7182	0.7717

1 (S) *Salmonella* Newport was inoculated to achieve 10^4^ colony-forming units (CFU)/mL of incubation fluid at the beginning of the incubation period.

2 N (Nitrate) concentrations at the beginning of the incubation period, when administered alone or in combination with tungstate, were 11.36 ± 2.28 μmol/mL (mean ± SD).

3 T (Tungstate) was supplemented as sodium tungstate dihydrate to achieve 100 mM at the beginning of the incubation period.

4 NA, Not applicable.

^a,b^Least squares means within rows with unlike superscripts differ at *p* < 0.05 based on a Tukey HSD All-Pairwise Comparisons Test.

The reductions in equivalents consumed during the production of methane, gaseous hydrogen, and VFA, and in the amounts of nitrate and nitrite metabolized, are presented in Table 4. A main effect of nitrate supplementation was observed in the amount of reducing equivalents consumed by methane production (*p* ≤ 0.01), with the nitrate-supplemented populations consuming 75% less of H_2_ than the untreated control populations (26.18 vs. 105.87 μmol H_2_/mL, respectively). There was also an effect of nitrate supplementation by tungstate treatment interaction (*p* = 0.05) such that reducing equivalents consumed by control populations containing no added nitrate or tungstate consumed more reducing equivalents (119 μmol H_2_/mL) than populations treated with tungstate (92.34 μmol H_2_/mL) or populations supplemented with nitrate, alone or with tungstate (25.53 and 26.83 μmol H_2_/mL, respectively). Consumption of reducing equivalents via methane production was not affected by other main or interaction effects (*p* ≥ 0.06).

A main effect of S. Newport inoculation was observed on hydrogen gas produced by the mixed rumen microbiota (*p* < 0.01), with *S*. Newport-inoculated populations producing more hydrogen gas than the non-inoculated populations (0.12 vs. 0.04 μmol H_2_/mL, respectively). No other main effects or interactions were observed on reducing equivalents consumed by hydrogen production (*p* ≥ 0.24).

**Figure 4:**
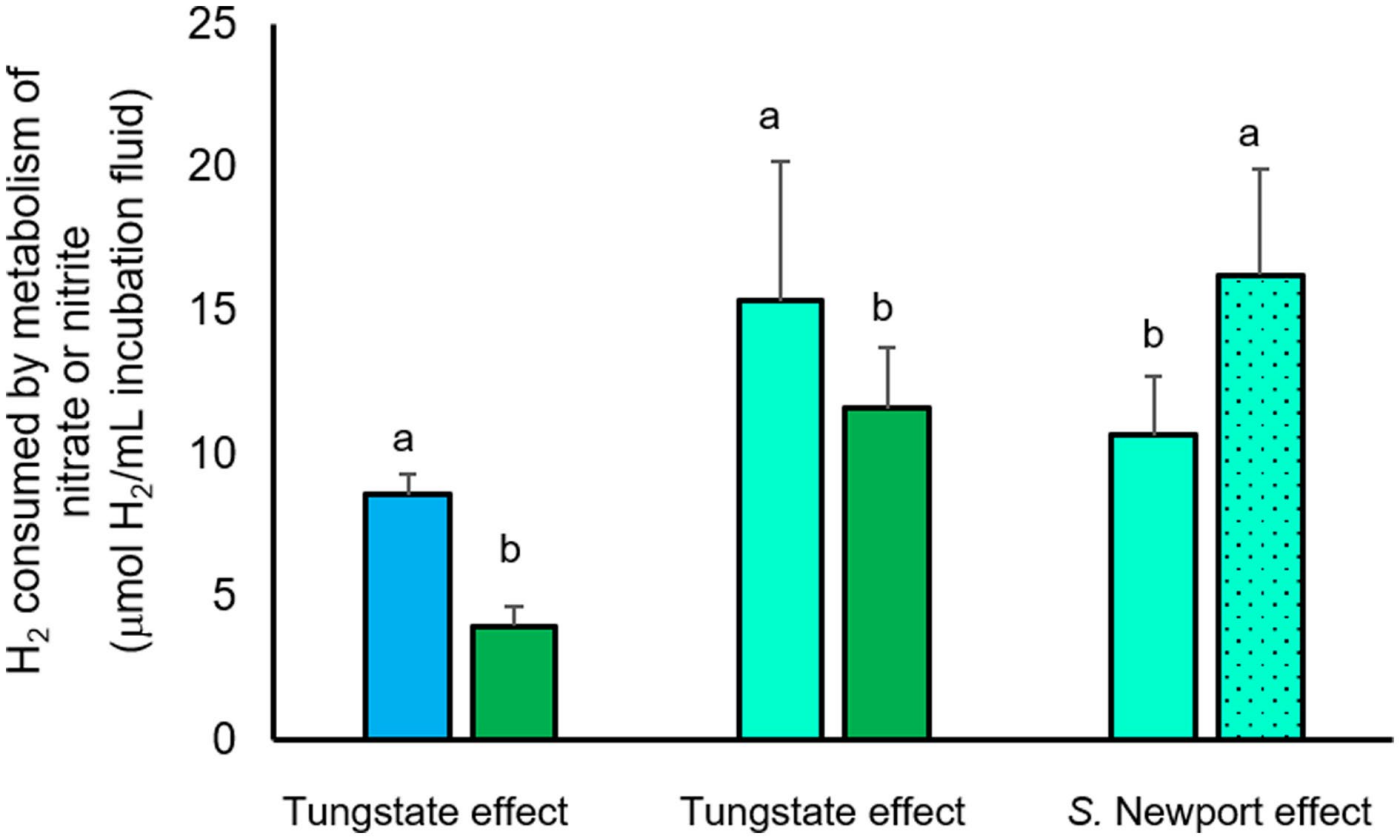
Least squares means ± standard errors for main effects of 100 mM tungstate treatment (green-filled bars) and *S*. Newport inoculation (spot-filled bars) on the consumption of reducing equivalents by nitrate (blue-filled bars) or nitrite (turquoise-filled bars) metabolized after 26-h incubation of mixed populations of ruminal microbes. White-filled bars represent non-treated controls. Least squares means associated with unlike lowercase letters differ at *p* < 0.05 based on a Tukey HSD All-Pairwise Comparisons test.

**Figure 5:**
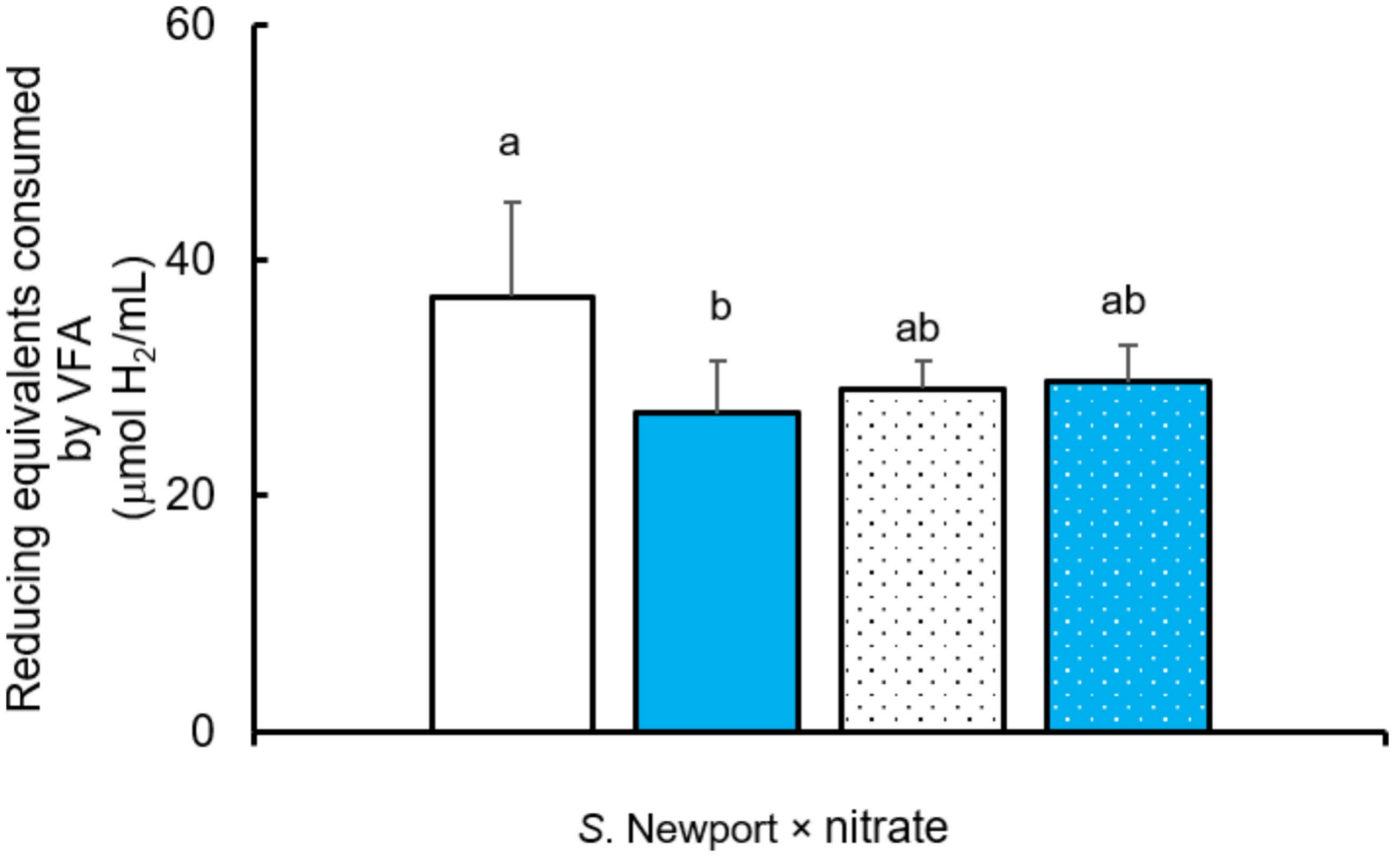
Least squares means ± standard errors for an interaction between *S*. Newport inoculation (spot-filled bars) and 10 mM nitrate supplementation (blue-filled bars) on stoichiometric estimates of reducing equivalents consumed by the production of volatile fatty acids (VFA) after 26-h incubation of mixed populations of ruminal microbes. White-filled bars represent non-treated controls. Least squares means associated with unlike lowercase letters differ at *p* < 0.05 based on a Tukey HSD All-Pairwise Comparisons test.

A main effect of nitrate supplementation (*p* = 0.04) (32.99 vs. 28.34 μmol H_2_/mL for nitrate-supplemented vs. unsupplemented populations, respectively) as well as an *S.* Newport by nitrate supplementation interaction (*p* = 0.02; Figure 4) were observed on reducing equivalents consumed via VFA production. However, no other main effects or interactions were observed (Table 4). A main effect of tungstate treatment was observed on reducing equivalents consumed by nitrate metabolism (*p* ≤ 0.01), and main effects of *S.* Newport inoculation and tungstate treatment (*p* ≤ 0.01) were observed on amounts of reducing equivalents consumed by nitrite metabolism (Figure 5), with consumption being decreased by nitrate and tungstate, respectively. No other main effects or interactions were observed on the consumption of reducing equivalents by nitrate or nitrite metabolism (*p* ≥ 0.08).

**Figure 6:**
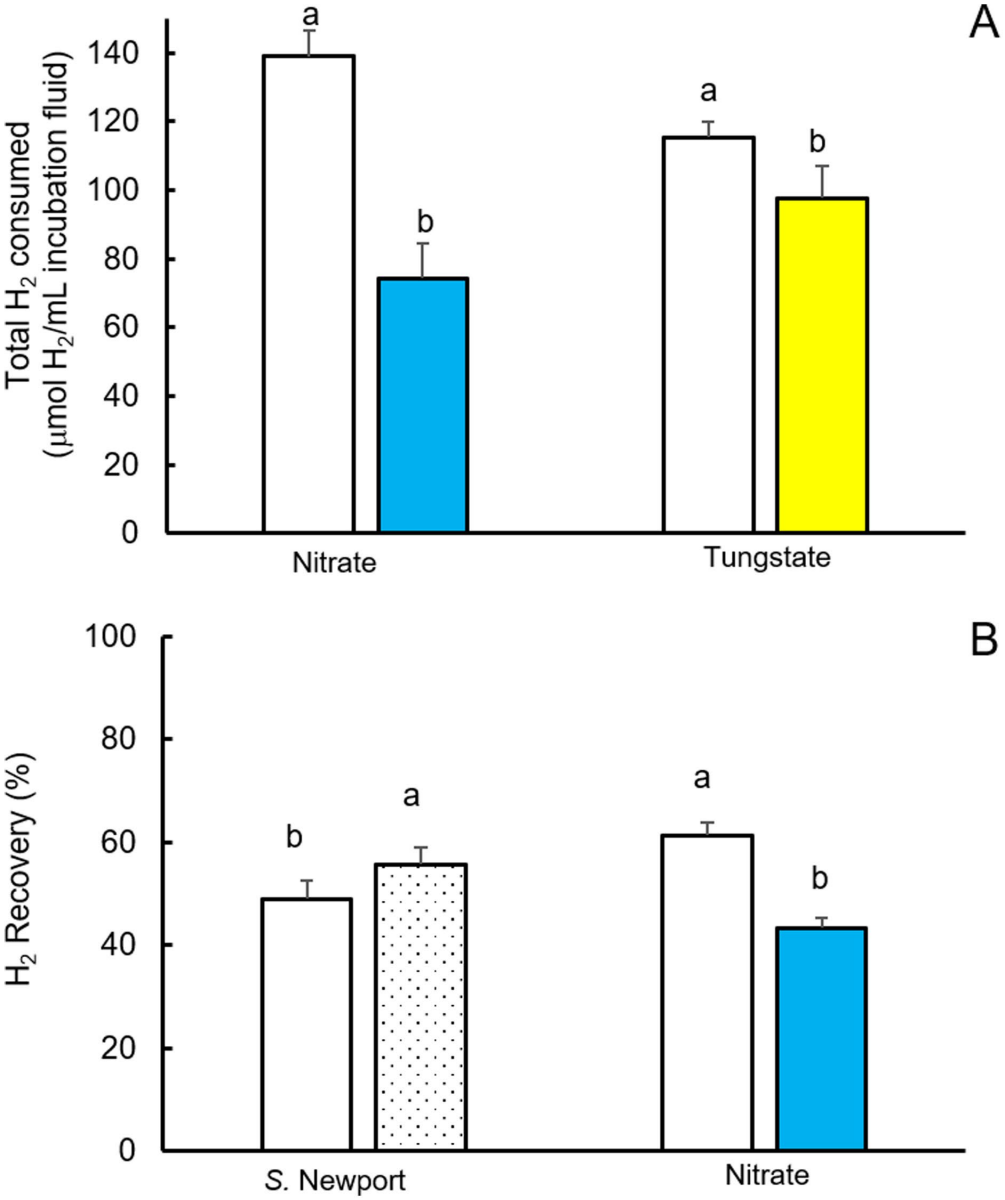
Least squares means ± standard errors for the main effects of 10 mM nitrate supplementation (blue-filled bar) and 100 mM tungstate treatment (yellow filled bar) on reducing equivalents consumed **(A)** and main effects of *S*. Newport inoculation (spot-filled bar) and nitrate addition on percent total recovery of reducing equivalents **(B)** after 26-h incubation of mixed populations of ruminal microbes. White-filled bars represent non-treated controls. Least squares means associated with unlike lowercase letters differ at *p* < 0.05 based on a Tukey HSD All-Pairwise Comparisons test.

Estimates of amounts of total reducing equivalents consumed varied considerably, ranging from 57.96 to 157.06 μmol H_2_ equivalents/mL and were affected by nitrate addition (*p* ≤ 0.01) and tungstate treatment (*p* = 0.03; Figure 6A). Percent recovery of reducing equivalents was positively affected by *S.* Newport inoculation (*p* = 0.01) and negatively affected by nitrate addition (*p* ≤ 0.01; Figure 6B), but other treatment or interaction effects were not observed.

## 4 Discussion

As reviewed by Latham et al. (2016), abundant evidence exists for the enrichment of nitrate-metabolizing bacteria within rumen microbiota; however, previous studies have focused mainly on indigenous nitrate-metabolizing bacteria, with few studies reporting the potential nitrate-associated enrichment of *Salmonella* within rumen populations (Correa et al., 2017; Anderson et al., 2026). Similarly, although tungstate inhibition of nitrate metabolism by pure and mixed populations of rumen microbes has been studied (Enoch and Lester, 1972; Gates et al., 2003; Korzeniowski et al., 1980, 1981; Prins et al., 1980; Marais et al., 1988; Takahashi et al., 1989), most of these studies reported effects on nitrate-metabolizing activity, with few reporting the effects of tungstate on rumen bacterial growth.

The nitrate-associated enrichment of *S.* Newport observed in the present study is consistent with *in vitro* findings by Correa et al. (2017) and the more recent study by Anderson et al. (2026) and may have food safety implications for animals fed supplemental nitrate. For instance, food-producing animals acquire *Salmonella* infections predominantly via oral ingestion; consequently, the rumen is a major compartmental reservoir occupied by *Salmonella*. Accordingly, dietary factors affecting the proliferation of *Salmonella* and their colonization in the rumen would be expected to influence how much of the pathogen passes to the lower gastrointestinal tract, where it has the opportunity to translocate and systemically infect the host. Whether dietary nitrate supplementation to well-fed ruminants would necessarily affect long-term carriage of *Salmonella* within the rumen or, ultimately, within the lower gastrointestinal tract is not yet known and warrants further investigation with live animals under production settings. Such studies could potentially allow for the determination of the effect of supplemental nitrate on total tract colonization by *Salmonella*, their adherence to intestinal mucosa, and ultimately their systemic translocation or long-term shedding in the animal.

The absence of appreciable proliferation of *S.* Newport or wild-type coliforms within the control rumen microbiota populations incubated without added nitrate or tungstate was not unexpected and suggests that washout of the pathogen may occur under *in vivo* conditions. In well-fed animals with a functionally mature rumen, for instance, the rumen environment is typically inhospitable for *Salmonella* due to the abundance of competitive amylolytic bacteria and their production of inhibitory factors such as acidic fermentation products (Brownlie and Grau, 1967; Chambers and Lysons, 1979; Mattila et al., 1988; Rasmussen et al., 1993). Consequently, upon ingestion by ruminants, *Salmonella* may pass transiently through pre-gastric compartments to successfully colonize the animal’s lower gastrointestinal tract. Upon arrival in the small intestine, the invading *Salmonella* elicits an inflammatory response by the host’s epithelial cells, which may induce intestinal-derived nitrate production capable of sustaining enhanced growth and colonization of *Salmonella* (Vázquez-Torres and Bäumler, 2016; Miller et al., 2022). In these cases, *Salmonella* passing from the rumen may ultimately become established and enriched within localized microenvironments near the gut mucosa (Winter et al., 2013; Vázquez-Torres and Bäumler, 2016; Miller et al., 2022).

Realizing that the ability to metabolize nitrate is a virulence trait of nitrate-respiring pathogens, Zhu et al. (2018) hypothesized and subsequently demonstrated that tungstate, an inhibitor of molybdenum-containing coenzymes involved in nitrate metabolism, reduced the competitiveness of select nitrate-respiring Enterobacteriaceae during *in vitro* culture and in experimentally challenged mice. Moreover, Yang et al. (2023) also reported tungstate-associated inhibition of *E. coli* grown in pure culture or co-culture with *Bacillus coagulans*. Conversely, in the present study, enhanced, rather than inhibited, growth of experimentally inoculated *S.* Newport and wild-type coliforms in anaerobic incubations of freshly collected ruminal microbiota was associated with tungstate treatment, which agrees with results from a recently published study conducted under similar conditions (Anderson et al., 2026). In both the present and recent studies (Anderson et al., 2026), an increase in *S.* Newport and wild-type coliforms was associated with the 100 mM tungstate treatment, whether alone or in combination with 10 mM nitrate, of more than 3.5 log units relative to untreated controls. As suggested earlier (Anderson et al., 2026), it is reasonable to hypothesize that the administration of tungstate, which is bound to four oxygen atoms, may have provided a source of reducible oxygen to support respiration. In support of this hypothesis, nitrate metabolism after 26 h of incubation of the ruminal populations was inhibited by tungstate treatment relative to untreated controls, indicating that molybdenum-associated enzymes related to nitrate metabolism were likely inhibited here and in earlier studies (Anderson et al., 2026). Moreover, when tested against pure cultures of *S.* serovars Newport, Dublin and Typhimurium, the 50 mM tungstate treatment similarly increased the growth of these bacteria while concurrently inhibiting nitrate metabolism; yet cultures of *S.* Newport treated with tungsten, in place of tungstate, effectively inhibited growth of the pathogen (Anderson et al., 2026).

Our finding that lactic acid bacteria, which are generally not considered competent in nitrate metabolism, were increased in the tungstate-treated populations by more than 0.4 log units compared to populations not treated with tungstate suggests that oxygen availability in the tungstate-treated populations may have contributed to their increased growth. Limitations of the present study, however, are that a single high dose (100 mM) of tungstate was added, and a limited number of bacterial populations were examined. For comparison purposes, however, the amount of tungstate added in the present experiment (100 mM), as well as the study of Anderson et al. (2026), was based on the higher amounts (up to 100 mM) reported to be effective against *E. coli* (Gates et al., 2003). Minimal effective doses used in some earlier studies against *E. coli* and other nitrate-metabolizing bacteria ranged from approximately 0.1 to more than 15 mM sodium tungstate (Enoch and Lester, 1972; Prins et al., 1980; Takahashi et al., 1989; Gates et al., 2003; Zhu et al., 2018). The high dose of tungstate used in this study may have exerted selective toxicity against other bacteria within the ruminal microbiota, thereby potentially facilitating the establishment of a competitive niche for *S.* Newport in their absence. Clearly, further research investigating multiple doses of tungstate and nitrate would be useful for elucidating potential effects under conditions that simulate the amounts that may be administered in practice.

Despite the unexpected tungstate-associated enrichment of *S.* Newport and the wild-type populations of coliforms and lactic acid bacteria observed in the present study and in the earlier study by Anderson et al. (2026), tungstate inhibited nitrate metabolism by mixed populations of ruminal microbes. However, rates of nitrate metabolism were only modestly reduced by tungstate, and the amounts of nitrate metabolized after 26 h incubation were also modestly decreased in the tungstate-treated populations. However, the ruminal populations tested in these studies were collected from a steer with no known prior exposure to nitrate; accordingly, likely, induction of optimal nitrate-metabolizing activity would not occur immediately. Peak accumulations of nitrite, a transient intermediate product of anaerobic nitrate metabolism, were observed by 6 h of incubation in populations of the previous study (Anderson et al., 2026), whereas peak accumulations occurred at the end of the 26-h incubation period in the nitrate-supplemented populations of the present study. This, however, was likely due to the higher nitrate dose (10 mM) administered in the present study than that administered (5 mM) in the earlier study (Anderson et al., 2026). As described earlier, a 10 mM nitrate dose achieved after meal consumption would equate to 8.2 g of nitrate ions per kg of dry matter intake if fed in two equal-sized meals per day. By comparison, Olijhoek et al. (2016) fed diets supplemented with low, medium, and high levels of nitrate (5.3, 13.6, or 21.1 g nitrate ion/kg of dry matter per day, respectively), offered in two equal-sized meals daily, to dairy cows (590 to 600 kg BW). Assuming a 120 L rumen volume as in the earlier example, these doses would achieve ruminal concentrations of 6.2, 17.1, and 26.2 mM, respectively, upon complete consumption of each meal, provided there is no residual or carryover of nitrate from earlier meals. Tungstate treatment inhibited nitrite consumption by ruminal microbial populations in both the previous and present studies. In the previous study (Anderson et al., 2026), results from the present study revealed a main effect of *S*. Newport on nitrite metabolism, with populations inoculated with S. Newport consuming 22% more nitrite than non-inoculated populations.

Total gas accumulations, as well as accumulations of hydrogen and methane, were within ranges typically observed for *in vitro* incubations, indicating the incubations were sufficiently anaerobic to support methanogenesis and anaerobic fermentation (Anderson and Rasmussen, 1998). A main effect of *S.* Newport inoculation, albeit modest, was observed on hydrogen accumulation in the present study. This is not unexpected as *Salmonella,* depending on conditions, can either produce or consume hydrogen or formate, both of which are substrates for methane production, in anaerobic environments such as that in the present study (Pinske and Sawers, 2016).

Populations of rumen microbes supplemented with nitrate, whether alone or in combination with tungstate, produced more than 70% less methane than unsupplemented populations, supporting previous research demonstrating that nitrate is a potent inhibitor of ruminal methanogenesis (Anderson and Rasmussen, 1998; Božic et al., 2009). Mechanistically, nitrate is recognized as an energetically favorable electron acceptor that can, at least partially, consume electrons within the rumen microbiota at the expense of methanogenic reduction of carbon dioxide to methane (Latham et al., 2016; Yang et al., 2016). Despite marked reductions in methane production observed by nitrate supplementation in the present study, the amounts of nitrate and nitrite consumed by ruminal microbiota could, at most, account for 13.3 to 27.6 μmol H_2_ equivalents, which translates to a decrease of only 3.3 to 6.9 μmol methane/mL or approximately 14 to 28% of the observed decrease in methane production.

For the non-*S.* Newport and *S.* Newport-inoculated populations supplemented with nitrate alone, 23 and 57% of the nitrite produced from the enzymatic reduction of nitrate were unmetabolized after 26 h of incubation, respectively, indicating that, under these experimental conditions, the incomplete metabolism of nitrite was not limited by the availability of reducing equivalents. In the rumen, nitrate and nitrite metabolism occur most rapidly at a neutral pH and, at pH 6.0 or lower, metabolism of nitrite produced from nitrate occurs at a slower rate, such that nitrite accumulates, and this is largely due to an adverse effect of decreasing pH on fermentation (Iwamoto et al., 2001; Latham et al., 2016). This could explain some, but not all, of the incomplete consumption of generated reducing equivalents by the nitrate-only-treated populations. Our finding that nitrate supplementation, in the absence of added *S.* Newport, was associated with decreased consumption amounts of reducing equivalents for the production of VFA is consistent with this observation. Additionally, it is likely that at least some of the generated reducing equivalents were consumed to support the log-fold increases in wild-type coliforms and experimentally inoculated *S.* Newport, as well as the lesser increases in lactic acid bacteria. Conversely, nitrate-supplemented populations co-treated with tungstate metabolized 95–99% of the available nitrite. However, the amount of nitrite produced by nitrate metabolism in the co-treated populations was considerably lower than that made available by nitrate-supplemented populations alone. Moreover, while not significant, amounts of reducing equivalents generated were appreciably low in the nitrate-supplemented and tungstate-treated populations, and amounts of reducing equivalents consumed were appreciably high enough in the *S.* Newport-inoculated populations to affect subsequent stoichiometric calculations of H_2_ recovery.

While a 53% tungstate-associated decrease in methane production was observed in the previous experiment of Anderson et al. (2026), methane production in the present study was only 17% lower in the tungstate-treated populations than the non-treated controls. The available data do not conclusively explain how tungstate mechanistically decreases methane production. However, it is possible that reducing equivalents otherwise destined for the reduction of carbon dioxide were consumed during the reduction of undefined oxygen moieties derived from tungstate. Alternatively, it is known that some acetogens (e.g., *Clostridium formioaceticum* and *Eubacterium limosum*) can incorporate tungsten into enzymes that reduce carbon dioxide, formate, or carboxylic acids to alcohols (Yamamoto et al., 1983; White and Simon, 1992), but the evidence for such is lacking here. Moreover, while certain rumen methanogens can express molybdenum- and tungsten-containing formyl-methanofuran dehydrogenases (Khairunisa et al., 2023), it is not known if these populations may be influenced by the high dose of supplemental tungstate employed in this study.

Volatile fatty acid production in the present experiment was considerably higher than in the earlier study by Anderson et al. (2026), suggesting more robust fermentation, possibly due to a protective effect of tungstate on methane production by sustaining a more reduced environment. This suggests that tungstate may have altered the redox potential of the *in vitro* incubations. Data related to the effects of tungstate supplementation on the redox state of anaerobic media are not readily available and were not measured in this or the previous study (Anderson et al., 2026); consequently, this hypothesis has yet to be confirmed.

Net production of acetate, propionate, and butyrate was lower in populations inoculated with *S.* Newport compared to non-inoculated populations. The lower acetate accumulation associated with *S.* Newport inoculation relative to the other VFAs also contributed to stoichiometric estimates of total acid production and hexose consumption, which were likewise reduced, as would be expected if the carbon substrates were used to support anabolic retention of carbon for maintenance and growth during respiratory metabolism rather than fermentation. Effects of nitrate, tungstate, *S.* Newport, or their potential interactions observed on the net production of the less abundant fatty acids were modest. Fermentation efficiency, which is an estimate of the caloric energy in hexose being recovered as acetate, propionate, and butyrate (Chalupa, 1977), was highest in *S.* Newport-inoculated populations supplemented with nitrate alone, reflecting a favorable proportion of acetate, propionate, and butyrate produced per amount of hexose fermented.

Consistent with results from the earlier study (Anderson et al., 2026), ammonia accumulation in the present study was higher in the nitrate-supplemented populations and, while not necessarily significant, was higher in the *S.* Newport-inoculated populations than in those not inoculated with *S.* Newport. Ammonia is considered the main, albeit not exclusive, end product of nitrite metabolism within populations of ruminal microbes (Kaspar and Tiedje, 1981). Consequently, our finding that ammonia accumulation in populations treated with nitrate was at most 1.27 μmol/mL above the accumulations measured in populations not treated with nitrate was unexpected. Accordingly, it seems reasonable to hypothesize that some of the metabolized nitrate may have been converted to nitrogen oxides or dinitrogen gas, as Latham et al. (2016) reported that up to 3.4% of administered nitrate was accounted for as nitrous oxide that had accumulated within populations of ruminal microbiota. Moreover, while Enterobacteriaceae, such as *Salmonella* and *E. coli,* are not considered major denitrifying bacteria, membrane-bound nitrate reductase (Nar), contributing to the conversion of nitrate to nitrite, has been reported to further convert nitrite to nitric oxide under some conditions (Gilberthorpe and Poole, 2008; Mills et al., 2008). This activity is thought to be associated with nitrite reduction catalyzed by flavorubredoxin (NorV) and cytochrome c nitrite reductase (NrfA) (Gilberthorpe and Poole, 2008; Mills et al., 2008). Alternatively, some of the nitrite-derived ammonia may have been assimilated and used for protein synthesis to support the proliferation of *S.* Newport, wild-type coliforms, and lactic acid bacteria that were observed in the nitrate-supplemented and tungstate-treated populations.

## 5 Conclusion

Under the conditions of the present study, nitrate supplementation of bovine rumen microbiota successfully mitigated methane production but was also associated with enrichment of an experimentally inoculated *S.* Newport. Contrary to our expectations, tungstate treatment promoted proliferation of the experimentally inoculated *S.* Newport as well as wild-type populations of coliforms and lactic acid bacteria, thus disproving the concept that tungstate, at the concentration used here, would unambiguously inhibit the growth of nitrate-respiring bacteria. Results also indicated that the tungstate effects were not limited to nitrate-respiring bacteria and suggest, but do not prove, a potential competition between lactic acid bacteria and *S.* Newport in the presence of tungstate.

At the concentrations used in this study, it seems reasonable to hypothesize that tungstate, which is bound to four oxygen atoms, could have served as an electron acceptor, either directly or indirectly, as a source of liberated oxygen, thereby supporting energy conservation in the present incubations. While it seems counterintuitive, it is possible that higher amounts of tungstate used in this study, compared with those in most earlier experiments, may have contributed to the differential results regarding bacterial growth. Conceptually, the lower tungstate doses used in some earlier studies (≤ 15 mM) may have been sufficient to inhibit energy conservation by nitrate respiration but inadequate to provide sufficient oxygen to support appreciable growth via oxygen respiration. This hypothesis, of course, remains to be tested. Still, the results from the present study indicate further research is needed to understand the potential for *Salmonella* enrichment and its implications for food safety in ruminants fed nitrate and to investigate conditions and doses of tungstate that may ultimately achieve safe and efficacious gastrointestinal control of *Salmonella*. Limitations of the present study include the use of single high doses of tungstate and nitrate, the high *S.* Newport inoculum, and the lack of metagenomic and transcriptomic analyses of the populations obtained from a single donor animal. Future studies incorporating molecular-based population studies and/or measurements of oxygen release, consumption, redox potential, and the toxic effects of tungstate or its potential metabolites in ruminal populations collected from multiple animals, ultimately conducted *in vivo*, would provide useful, functional ecosystem characterizations.

## Data Availability

The raw data supporting the conclusions of this article will be made available by the authors, without undue reservation.
